# A Policy-into-Practice Intervention to Increase the Uptake of Evidence-Based Management of Low Back Pain in Primary Care: A Prospective Cohort Study

**DOI:** 10.1371/journal.pone.0038037

**Published:** 2012-05-25

**Authors:** Helen Slater, Stephanie Joy Davies, Richard Parsons, John Louis Quintner, Stephan Alexander Schug

**Affiliations:** 1 School of Physiotherapy, Curtin Health Innovation Research Institute, Curtin University, Perth, Western Australia, Australia; 2 Pain Medicine Unit, Fremantle Hospital and Health Service, Perth, Western Australia, Australia; 3 School of Occupational Therapy and School of Pharmacy, Curtin Health Innovation Research Institute, Curtin University, Perth, Western Australia, Australia; 4 Pharmacology and Anaesthesiology Unit, University of Western Australia, Perth, Western Australia, Australia; 5 Department of Anaesthesia and Pain Medicine, Royal Perth Hospital, Perth, Western Australia, Australia; Linkoping University, Sweden

## Abstract

**Background:**

Persistent non-specific low back pain (nsLBP) is poorly understood by the general community, by educators, researchers and health professionals, making effective care problematic. This study evaluated the effectiveness of a policy-into-practice intervention developed for primary care physicians (PCPs).

**Methods:**

To encourage PCPs to adopt practical evidence-based approaches and facilitate time-efficient, integrated management of patients with nsLBP, we developed an interdisciplinary evidence-based, practical pain education program (gPEP) based on a contemporary biopsychosocial framework. One hundred and twenty six PCPs from primary care settings in Western Australia were recruited. PCPs participated in a 6.5-hour gPEP. Self-report measures recorded at baseline and at 2 months post-intervention included PCPs' attitudes, beliefs (modified Health Care Providers Pain and Impairment Relationship Scale (HC-PAIRS), evidence-based clinical practices (knowledge and skills regarding nsLBP management: 5-point Likert scale with 1 =  nil and 5 =  excellent) and practice behaviours (recommendations based on a patient vignette; 5-point Likert scale).

**Results:**

Ninety one PCPs participated (attendance rate of 72%; post-intervention response rate 88%). PCP-responders adopted more positive, guideline-consistent beliefs, evidenced by clinically significant HC-PAIRS score differences (mean change  = −5.6±8.2, p<0.0001; 95% confidence interval: −7.6 to −3.6) and significant positive shifts on all measures of clinical knowledge and skills (p<0.0001 for all questions). Self management strategies were recommended more frequently post-intervention. The majority of responders who were guideline-inconsistent for work and bed rest recommendations (82% and 62% respectively) at pre-intervention, gave guideline-consistent responses at post-intervention.

**Conclusion:**

An interprofessional pain education program set within a framework that aligns health policy and practice, encourages PCPs to adopt more self-reported evidence-based attitudes, beliefs and clinical behaviours in their management of patients with nsLBP. However, further research is required to determine cost effectiveness of this approach when compared with other modes of educational delivery and to examine PCP behaviours in actual clinical practice.

## Introduction

For patients and primary care physicians (PCPs) alike, persistent non specific low back pain (nsLBP) is well recognised as a “heart sink” [1] and the label nsLBP covers up our almost complete ignorance of underlying pain mechanisms. Critically, the management of patients with nsLBP is unsatisfactory and the resultant burden of disease at both individual and societal levels has become significant [2], [3], [4]. Models of care, service delivery and individual practitioner practices in the assessment and management of nsLBP also vary [5], [6] further impacting negatively upon patient outcomes [7]. Barriers to the implementation of current evidence-informed best practice in primary care have been identified [8], [9], [10] and, in parallel, patients continue to seek a pathway towards less pain and disability [11].

Despite the significant and escalating costs incurred to health systems from the use of various investigational and imaging studies [12] related to diagnosis and management [13], [14] and the costs associated with co-morbid pain [15], clinical outcomes for patients have not improved [16]. Additional contributors to poor patient outcomes include a lack of adherence to guidelines [17], [18] or lack of knowledge by practitioners [19] of best practice guidelines [20], [21] and the variable translation of evidence into practice in the primary care setting [22]. Heterogeneity in nsLBP cohorts [23], [24], [25] compounds these issues, although novel protocols to subgroup patients are emerging [17], [26]. Different systems of health care delivery complicate data comparisons and interpretation of trial outcomes is dogged by a lack of standardised pain assessment measures [27]. System inefficiencies exist due to inadequate use of validated screening criteria designed to match patient complexity with an appropriate level of resource allocation [28]. Furthermore, as there is no simple biomarker for pain and the lived experience cannot be inferred from imaging studies of the spine, including computed and functional magnetic resonance tomography, consistent adoption of evidence-based practice appears even more critical in achieving positive health and economic outcomes [22].

By contrast, the adherence to guidelines by practitioners and the adoption of appropriate self management practices by patients are currently considered rational strategies to reduce the burden of spinal pain [28], [29], [30] and to deliver cost effective patient outcomes [18], [22]. Previously we have demonstrated reduced wait-times and costs at a public pain medicine unit and increased use of active pain management strategies [30] following a system redesign from a traditional model to one that delivers interprofessional patient group education sessions prior to individual appointments [28]. Interprofessional care, that is care arising from the provision of comprehensive health services to patients by multiple health caregivers who work collaboratively in order to deliver quality care evidence-based care within and across settings, is associated with a reduction in health care utilisation and improved function [30], [31], [32]. Integrating biomedical pain management strategies with cognitive behavioural approaches in a health service delivery model such as ours perforce uses an interprofessional platform [28], [30], that combines evidence-based guidelines [33] pharmacological [34], [35] and interventional procedural options [36] with self-management training for patients [30], [37], education in the neurophysiology of pain [38], [39], active strategies such as pacing (time-contingent graded activity [39]), moderating fear avoidance behaviours [40] and active movement-based strategies [41], [42]. In primary care practice, adding advice, education and exercise, or exercise and behavioural counselling to usual physician care, is also more cost-effective than usual physician care alone [32]. However, most physicians have received limited training to cope with the multidimensional nature of complex pain. The implementation of such interprofessional evidence-based models would appear to require a continual cycle of education coupled with practical skills, delivered both to consumers and health providers as described in the Western Australian, Department of Heath, Spinal Pain Model of Care [43]. Therefore, to better align policy and practice [28], [30] in Western Australia, we developed, implemented and evaluated the effectiveness of such an interprofessional educational program designed to enhance the knowledge and skills of primary care physicians (PCPs) managing patients with nsLBP.

## Methods

### Subjects

#### Study design/population

Using a prospective cohort study design, PCPs from metropolitan Perth, Western Australia were invited to attend the General Practitioner Pain Education Program (gPEP). This program was designed to upskill PCPs with practical evidence-based management of patients with nsLBP. Inclusion criteria required that PCPs were registered and practising in primary care. Exclusion criteria included PCPs or specialists already working in a multidisciplinary team that treated patients with acute and chronic musculoskeletal pain.

#### Ethics statement

The study was approved by the local South Metropolitan Area Health Service, Fremantle Hospital and Health Service, Department of Health (Government of Western Australia), Human Research Ethics Committee (no. 08/371) and adhered to the Code of Ethics of the Declaration of Helsinki. Written informed consent was obtained from all participants. The Royal Australian College of General Practitioners (RACGP) accredited gPEP as a Continuing Professional Development activity attracting a Category 1 rating with the maximum of 40 points awarded to practitioners post-completion of required activities.

#### Participation, consent and anonymity

**Figure 1:**
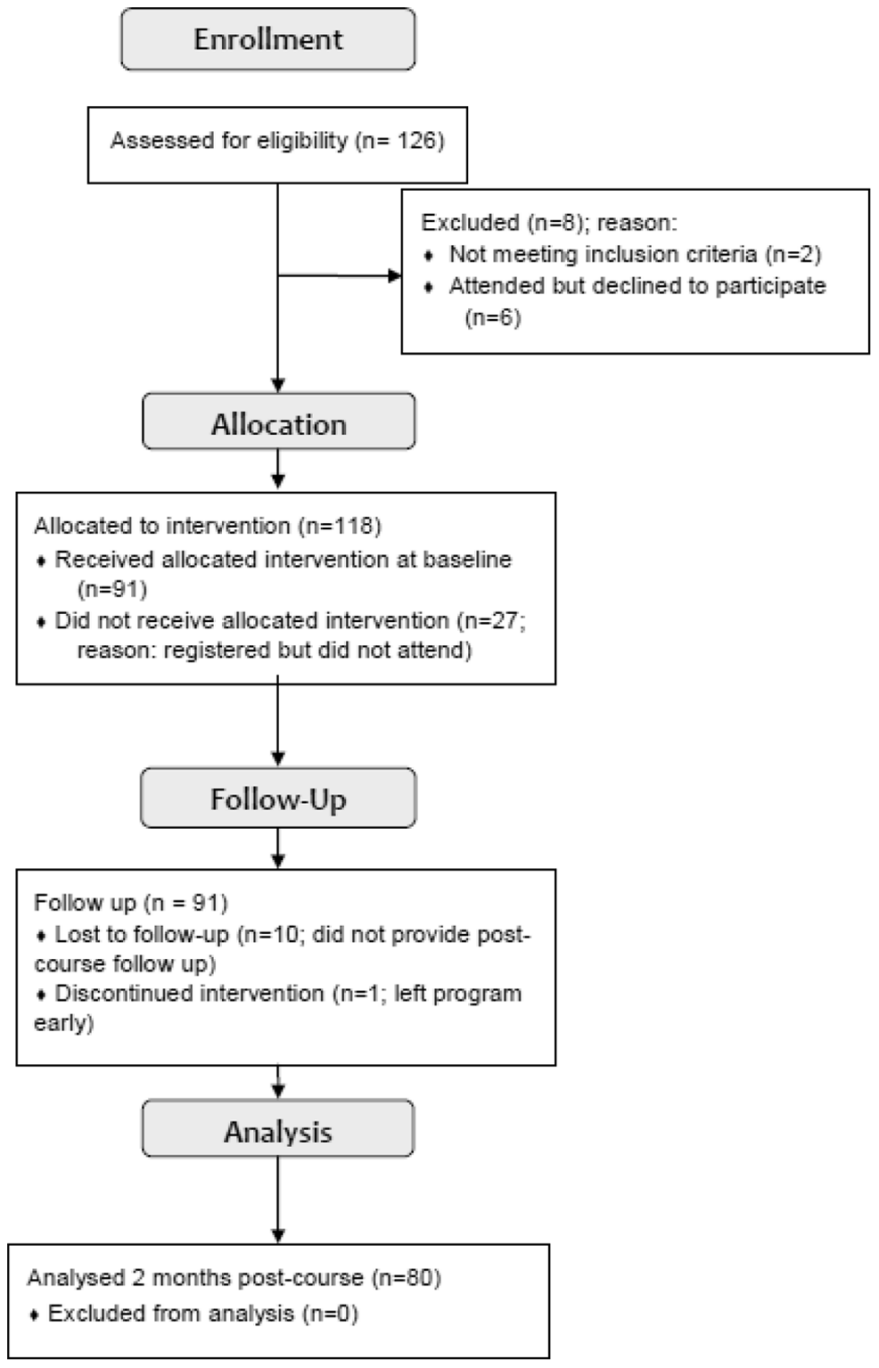
This flow chart indicates the study recruitment process. Note that some physicians registered and attended but were not included in the analyses as they did not consent to their data being used. These participants were still eligible for their maximum continuing education points if they submitted their pre and post course questionnaires.

PCPs were invited through two nominated metropolitan General Practitioner Networks (GPNs) to register and attend one of five workshops, which were run consecutively over a five month period in 2009. One hundred and twenty six PCPs were registered for gPEP (Figure 1). Of this number, eight were excluded: two did not meet the inclusion criteria (not currently practising); six met the inclusion and attended gPEP but did not consent to use of the data for the purposes of the study. Of the one hundred and eighteen remaining registered PCPs, ninety one attended and participated, with the twenty seven remaining registrants not attending on the day. All registrations were accepted by the two geographically separate, metropolitan GPNs. These GPNs allocated a pre-selected unique study identification code to each of the registered PCPs. A set of these unique study codes was pre-allocated by the research team to each GPN. This unique code appeared on all registrants' subsequent data sets and on the associated GPN databases allowing for the efficient cross-matching of both responders and non-responders throughout the study, while also maintaining participant confidentiality. One researcher (HS) was also a member of the educational team and was aware of the unique study identification codes. This researcher was not involved in the data entry or analysis. The remainder of the educational team was blind to the data collection, entry and analysis.

On the day and immediately prior to the intervention, written consent forms were completed by attending participants and collected. Participants were then instructed to open an envelope containing their uniquely-coded data set. This data set contained a battery of questionnaires, as fully outlined below. Once completed, each participant sealed their baseline data in the individual envelopes provided. Data sets were immediately collected by the research team and attending GPN representatives. Following each of the educational programs, one investigator (HS) cross-matched the registration list with the final attendance list and with the signed consent forms. This enabled identification of participants who registered but did not attend, or who did attend but did not consent to their data being used for the purposes of this study. For those participants (n = 6) who did attend the program, but did not consent to the study, no data were included in any analysis. However, for these participants, data were still collected as completing the battery of questionnaires was a requirement for awarding of continuing education points. At 2 months post-intervention, each GPN mailed out to their allocated participants, a code-matched post-course data set with instructions to complete and to send back in the pre-paid envelope or to fax back to the GPNs. Each participant data set was then matched and logged against their baseline data set. In this way pre- and post-intervention data could be matched at data entry. Non-responders were contacted only by the GPNs, who requested completion of the post-course questionnaires to satisfy RACGP post course activity requirements.

#### Intervention: Educational Team and Educational Materials

The interprofessional educational team were all actively engaged in either tertiary facilities (hospitals providing specialised consultative health care, and requiring a referral from a primary or secondary health care facility) and/or private practice non-hospital primary care and hospital-based pain management facilities and/or university facilities and who worked together in various clinical and research combinations across these facilities. The team included four pain medicine specialists (one of whom was also a rheumatologist), one senior occupational therapist, four senior postgraduate-qualified musculoskeletal physiotherapists and two clinical psychologists. To ensure the educational content was closely aligned with and relevant to a primary care setting, external feedback was also sought from and provided by PCPs including representatives of the RACGP and the GP networks, and a clinical academic PCP.

**Figure 2:**
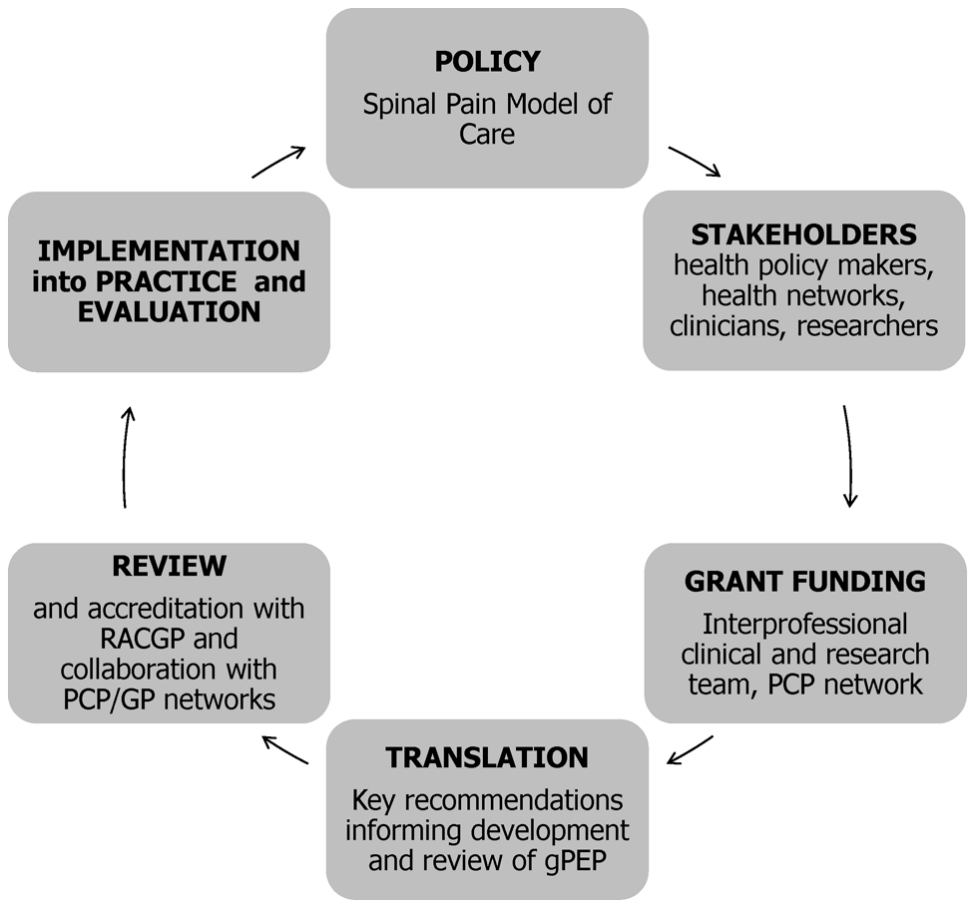
The framework involved in developing and implementing the gPEP intervention is described in this schematic. The Western Australian Spinal Pain Model of Care (MOC) is a policy document which describes a framework on which evidence can be implemented into policy and practice to ensure consumers with spinal pain receive the ‘right’ care, at the ‘right’ time, from the ‘right’ team and in the ‘right’ place. The Spinal Pain MOC addresses key gaps in policy and practice. Starting with the Model of Care, key stakeholders interested in spinal pain collaborated to apply for grant funding. Key recommendations from the MOC informed the focus of the educational content for gPEP, and the content was peer reviewed (including GP network engagement) and accredited through the Royal Australian College of General Practitioners (RACGP) for Continuing Professional Development points. The intervention was then implemented and the effectiveness evaluated.

The implementation framework for this intervention is summarised in Figure 2 and was based on ‘The Western Australian Spinal Pain Model of Care’ [43] with a focus on key recommendations 1–4 (p. 8 and p. 33 of the Model of Care; http://www.healthnetworks.health.wa.gov.au/modelsofcare/docs/Spinal_Pain_Model_of_Care.pdf). Five members of the educational team, 3 of whom also co-authored this study (HS, SD, JQ), contributed to the development of this evidence-based Model of Care which was designed to ensure consumers with spinal pain receive the ‘right’ care, at the ‘right’ time, from the ‘right’ team and in the ‘right’ place.

**Figure 3:**
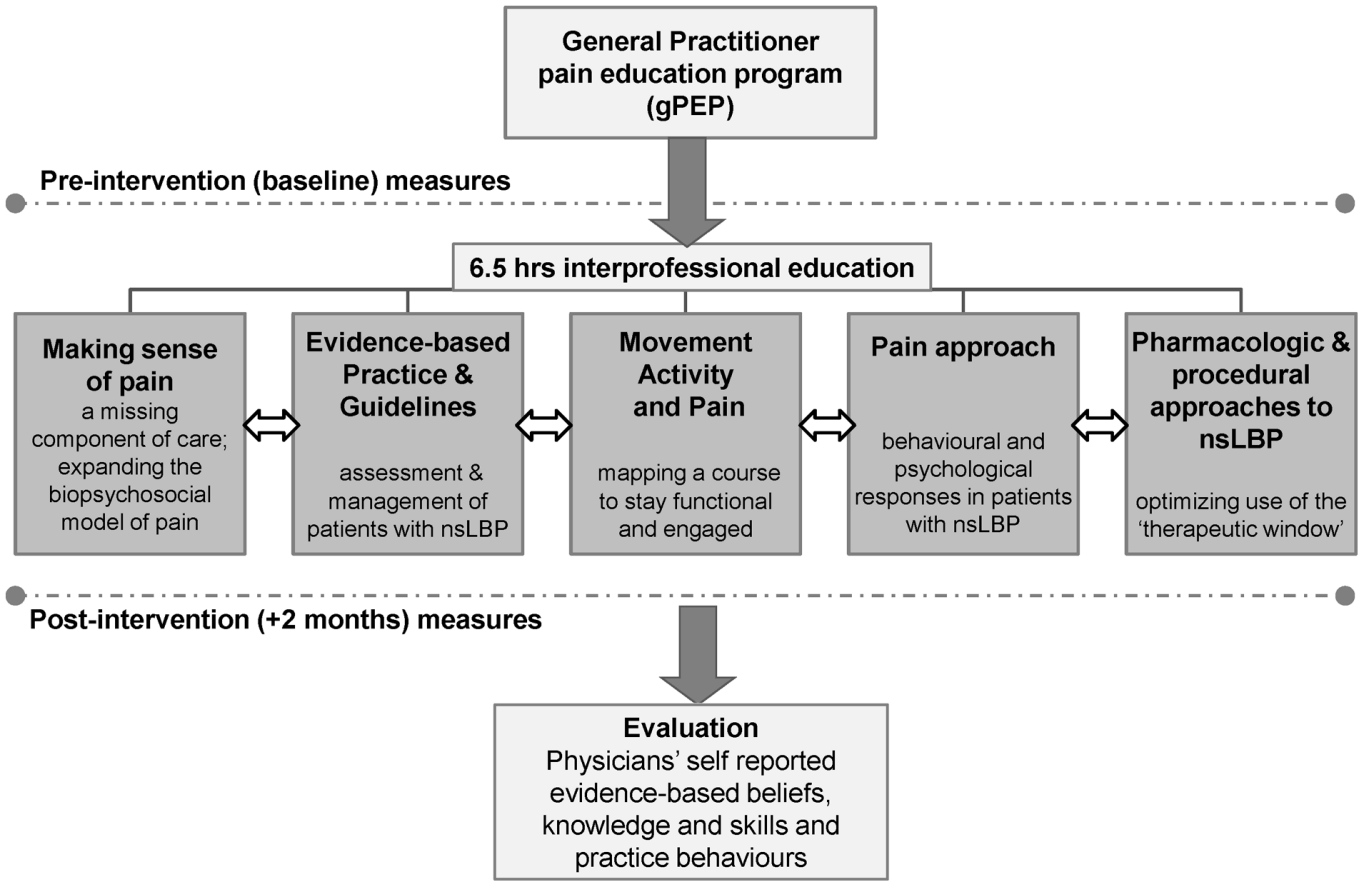
The interprofessional model of low back pain education for primary care physicians, is shown. Physicians' evidence base knowledge and skills and clinical practice behaviours were measured at baseline (upper dotted line) and at 8 weeks post intervention (lower dotted line). Five modules were presented over a single day. Each of 5 modules was presented with a short evidence based lecture of 15–20 minutes duration and was accompanied by a related case study integrating and applying the relevant clinical knowledge and skills. Each case study was designed to facilitate interprofessional engagement between both PCPs and the educational team, so participant groups were limited in size (typically n≤12) with each comprising a micro-interprofessional team (pain medicine specialist, clinical psychologist, physiotherapist and occupational therapist). The horizontal arrows indicate the integration of evidence base between and across all modules. Case studies targeted clinical practice related to each module, but also included other modular information, as appropriate.

The educational team collaboratively developed and delivered the 6.5 hour single day gPEP program (Figure 3) which included the following modules: (1) Making sense of pain: a missing component of care; (2) Clinical guidelines and evidence-informed best practice for the assessment and management of patients with nsLBP; (3) Movement, activity and pain; pacing activity and goal setting: helping patients with nsLBP map a meaningful course through every-day life; (4) Response to pain: psychological and behavioural factors in managing patients with nsLBP; and (5) Pharmacologic and procedural approaches to the management of patients with nsLBP.

Each module comprised a 15–20≤ minute evidence-based, guideline-informed lecture which was followed by an action-learning [44], interactive, ‘know-do’ case study of 45–60≤ minutes duration. Each case study was presented as a patient vignette with clinically relevant questions and interactive discussions relating to the implementation of evidence into clinical practice, specifically targeting appropriate clinical practice behaviours and focusing on practical patient-oriented active self management strategies and co-care. These case-based studies were focused on a matching of resources and management approaches to the level of a consumer's pain and disability (that is, low pain and disability were approached using less complex approaches and high pain and disability were approached using more complex, in-parallel multimodal approaches). Screening tools (for example, Orebro [45], Depression, Anxiety and Stress Scale [46], painDETECT [47]) were presented and their application and scoring demonstrated with reference to these case studies, with 2 specific aims: (i) to provide busy PCPs with a time-efficient diagnostic triage system for screening patients with LBP; and (ii) to enable PCPs to match their clinical findings with scores from tools designed to reflect the multidimensional aspects of pain.

The clinical guidelines which informed the development of the educational materials for this study included: (i) the Australian Evidence-Based Management of Acute Musculoskeletal Pain: A guide for clinicians [48] (ii) the New Zealand Clinical Group Guidelines for the Assessment and Management of Acute Non-Specific Low Back Pain [49], [50]; (iii) the European Low Back Pain guidelines [51], [52], [53], [54] (iv) Diagnosis and treatment of low back pain: a joint clinical practice guideline from the American College of Physicians and the American Pain Society [55]. The updated National Institute for Health and Clinical Excellence guidelines for the management and assessment of nsLBP [56] were not available at the time of the program development, but once available were subsequently reviewed in 2009 to ensure there were no significant evidence omissions.

Program participants were provided with a hard copy of a workbook containing all lecture materials and case studies, a summary table of evidence for the management of patients with acute and chronic nsLBP and a CD with the clinical guidelines listed above and any guideline-associated patient information sheets. All participants were also offered access to an online, not-for-profit database (myLibrary) used as a sustainable repository for all course materials and updates. Two free, optional “web-labs” were also provided to up-skill participants in web-based evidence searches and storage of information using a previously developed evidence-based storage database (http://www.mylibrary.net.au/).

### Study protocol

#### Intervention Measures

The study protocol used for this trial was based in part on a protocol described by Evans et al [8]. A battery of quantitative measures including PCP's attitudes, beliefs, knowledge and practical skills and clinical practice behaviours regarding the assessment and management of people with nsLBP, was undertaken at baseline (pre-intervention, immediately prior to the intervention) and repeated at 2 months post-intervention. This 2 month post-intervention time frame was implemented to align with the RACGP accreditations for the awarding of Category 1 Continuing Professional Development points, which require that a reinforcing activity (here, the post course battery of questionnaires) be undertaken within 2 months of completion of the training programme. The awarding of points was a significant incentive to PCPs who require a minimum number of 130 professional development points (i.e.; ∼30% of the total) per triennial cycle. Additionally, the 2 month time frame allowed for 5 separate interventions to be completed (pre- and post-) within the 12 month project funding period.

#### Study instruments

The Health Care Providers Pain and Impairment Relationship Scale (HC-PAIRS) tool is a reliable and valid single factor measure [57] of health care providers' attitudes and beliefs about the relationship between back pain and impairment [57], [58]. A subsequent modification of HC-PAIRS [8] was most appropriate for use in the primary care context of our study, as this version specifically concerns the way in which low back pain affects physical function and the total score can serve as a predictor for work and activity recommendations. There are 13 items in this modified questionnaire [8], with the responses recorded on a 1–7 Likert scale (ranging from 1 =  ‘Complete disagreement’ to 7 =  ‘Complete agreement’). Responses are summed to form a total HC-PAIRS score, giving a possible range from 13 to 91. As items 1, 6 and 12, were positively worded, these responses were reverse-scored prior to analysis. The higher a respondent's score, the stronger is a belief that pain necessarily implies disability [59] and that low back pain should affect daily function [8]. Lower scores align better with current evidence regarding nsLBP and indicate a movement towards disagreement with the questions (which generally suggest that management of lower back pain should involve rest rather than activity).

PCPs were asked to use the gPEP questionnaire to self-rate their knowledge and skills in regard to the use of current evidence-based approaches to their patients with nsLBP (the full questionnaire is shown in results). These questions were developed by the interprofessional educational team and based on the related guidelines for LBP and on professional consensus. These questions reflected the current evidence-based knowledge and the practical pain management skills deemed necessary for PCPs in order to improve the management of patients with nsLBP. Responses to each question were graded on an ordinal scale of 1–5, ranging from 1 =  ‘Nil’; 2 =  ‘Minimal’; 3 =  ‘Acceptable’; 4 =  ‘Good’; to 5 =  ‘Excellent’ and were individually scored. Based on the clinical consensus of the interprofessional team, and in line with the clinical guidelines used in this study, a rating of 1 or 2 was taken to indicate clinically inadequate (and guideline-inconsistent) responses, while the remaining responses (3–5) were regarded as clinically adequate (and guideline-consistent). The frequency per week that a PCP would advise or assist their patients in a certain activity was rated as ‘1–5 times per week’; ‘6–10 times per week’; ‘more than 10 times per week’ or ‘Never’ (this questionnaire is shown in results). PCPs were also asked to list the three most important things they gained from participating in gPEP.

Using a previously described patient vignette [8] and based on questions originally documented by Rainville et al. [60], PCPs were questioned regarding their recommendations for activity, work and bed rest for a patient who was experiencing acute nsLBP. In summary, and with full acknowledgement to this component of the protocol, as described by Evans et al [8], this patient vignette described a 28 year old female with no dependents, presenting with a three week history of a first episode of nsLBP related to lifting at work. Her pain was localised to the low back, she could sit for ten minutes and walk for about 100 metres before pain levels stopped her, sleep was undisturbed and there was no evidence of any associated serious pathologies or neurological compromise. Examination indicated no neurological compromise and a negative straight leg raise, lumbar flexion was quite limited and provocative. She was anxious to return to work as a hospital cafeteria manager but pain was limiting her and she had not consulted any health professional since the onset of her nsLBP. The format chosen to capture responses to each of three case-related questions was a 5-point Likert-type scale, with a left to right scale progression indicating a progressively more active approach to activity and work and towards less bed rest. In accordance with the Evan's et al [8] protocol, guideline-consistent responses for each question were scored as follows: question 1 (4 and 5); question 2 (3, 4, and 5); question 3 (4 and 5). All other responses were classified as ‘guideline inconsistent’.

### Statistical analysis

Response rates were calculated by dividing the number of respondents with completed surveys by the total number of consenting participants at baseline and at post-course. Standard descriptive statistics were used to summarise the age and gender of the participants.

#### Attitudes and beliefs (HC PAIRS)

The 7-point ordinal scale was treated as a continuous scale for the purpose of analysis of HC-PAIRS scores. As we were interested in the impact of key guideline messages on specific aspects of beliefs and attitudes to LBP, for each item, the mean and standard deviation (SD) of the score was calculated at both pre- and post-intervention. The change in score was calculated along with its 95% confidence interval and the paired t-test was used to identify whether any statistically significant change had occurred. The same analysis was performed on the HC-PAIRS total score (sum of all item responses). A t-test was used to compare the mean HC-PAIRS scores at baseline between participants who were, and were not, classified as ‘guideline consistent’ (from the patient vignette).

#### Self-reported knowledge and skills (gPEP questionnaire)

Frequencies and percentages of clinically inadequate responses both pre- and post-intervention were tabulated. Formal assessment of the change in responses was performed using paired t-tests on the raw (un-categorised) responses. This analysis treated the ordinal (Likert scale) responses as measurements on a continuous scale, and identified the statistical significance of any mean change in score from baseline to follow-up. Responses to questions regarding the frequency of PCPs advising use of exercise, lifestyle changes, self management and co-ordinating patient care with other health professionals, were grouped into categories ‘Never’ and ‘at least once per week’. Frequencies and percentages of ‘Never’ responses were tabulated for responses obtained both pre- and post-intervention. Cohen's kappa statistic is usually used to measure agreement between measures taken at two different times or by two different observers, with a value between 0.75 and 1 indicating very strong agreement. Conversely, a low value of kappa indicates poor agreement, which in the present study would indicate that the intervention had made a significant impact on responses. Kappa was calculated for each of these items, and the change in responses from pre- to post-intervention are also tabulated.

PCPs were also asked to ‘list the 3 most important things you gained from participating in this course’. These items were collapsed into key themes and presented as a percent of the total number of responses.

#### Practice behaviour

The frequencies and percentages of ‘guideline-inconsistent’ responses for each of the 3 questions were tabulated pre- and post-intervention. The kappa statistic was used to assess the degree of change in responses comparing pre- to post-intervention.

## Results

Of the one hundred and eighteen PCPs registered, ninety one attended and participated (attendance rate of 72%). Demographic and clinical data are shown in Table 1.

**Table 1:** The demographic and clinical practice characteristics of primary care physicians (PCPs) participating in gPEP^†^.

Characteristic	Number	Mean (SD) [min – max]
Age (years)	64	51.6 (11.8) 29–77
Gender: Male	45/81 (55.6%)	
In your clinical practice(s), do you have access to interdisciplinary training and/or health professionals? (Yes responses: total n/N (%))	53/73 (72.6%)	
Do you have access to health professionals from other disciplines to assist a team approach to acute and chronic LBP management? [Yes responses: total n/N (%)]	61/73 (83.6%)	
PCPs accessing myLibrary ^¥^ n/N (%)	37/81 (45.7%)	

Data are expressed as Yes responses [n/N (%)] for categorical variables, and N, mean (SD) and range for continuous variables; ^†^ gPEP general practitioner pain education program; ^§^ LBP: low back pain; ^¥^ myLibrary (http://www.mylibrary.net.au/) is a not-for-profit database used as a sustainable repository for all course materials and evidence-based low back pain updates.

### Attitudes and Beliefs (Modified HC Pairs Questionnaire)

Mean (SD) scores are shown pre- and post-intervention, the 95% confidence interval for the difference in means and the p-value for each item of HC-PAIRS (Table 2). In addition, a total HC-PAIRS score was generated. The HC-PAIRS score difference (n  =  mean change  = −5.6±8.2, p<0.0001; 95% confidence interval: −7.6 to −3.6) demonstrated a clinically significant move towards disagreement with the questions, that is, against the suggestion that management of lower back pain should involve rest rather than activity.

**Table 2:** Primary care physicians' beliefs regarding low back pain and associated disability.

HC PAIRS items	Pre-intervention Mean (SD)	Post-intervention Mean (SD)	Difference Mean (95% CI)	p-value
1 **Low** back pain patients can still be expected to fulfil work and family responsibilities despite pain	2.6 (1.4)	2.2 (1.0)	−0.4 (−0.7 to −0.0)	0.0448
2 An increase in pain is an indicator that a **low** back pain patient should stop what they are doing until the pain decreases	3.8 (1.9)	3.1 (1.9)	−0.9 (−1.3 to −0.4)	0.0002
3 **Low** back pain patients cannot go about normal life activities when they are in pain	3.0 (1.9)	2.5 (1.5)	−0.5 (−1.0 to −0.1)	0.0244
4 If their pain would go away, **low** back pain patients would be every bit as active as they used to be	4.3 (1.8)	3.9 (2.1)	−0.5 (−0.9 to −0.1)	0.0253
5 **Low** back pain patients should have the same benefits as the handicapped because of their **painful** problem	2.5 (1.6)	2.3 (1.6)	−0.2 (−0.6 to 0.2)	0.2576
6 **Low** back pain patients owe it to themselves and those around them to perform their usual activities even when their pain is bad	4.5 (1.7)	4.6 (1.7)	0.2 (−0.3 to 0.6)	0.5340
7 Most people expect too much of **low** back pain patients, given their pain	3.3 (1.6)	3.0 (1.7)	−0.4 (−0.8 to 0.0)	0.0716
8 **Low** back pain patients have to be careful not to do anything that might make their pain worse	3.8 (1.9)	3.0 (2.0)	−0.9 (−1.4 to −0.5)	<0.0001
9 As long as they are in pain, **low** back pain patients will never be able to live as well as they did before	3.2 (1.8)	2.6 (1.8)	−0.6 (−1.0 to −0.2)	0.0030
10 **Low** back pain patients have to accept that they are disabled persons, due to their pain	1.9 (1.3)	1.7 (1.1)	−0.2 (−0.5 to 0.2)	0.3204
11 There is no way that **low** back pain patients can return to do the things that they used to unless they first find a cure for their pain	1.9 (1.3)	1.9 (1.4)	0.0 (−0.2 to 0.3)	0.9230
12 Even though their pain is always there, **low** back pain patients often don't notice it at all when they are keeping themselves busy	3.1 (1.6)	2.7 (1.4)	−0.2 (−0.6 to 0.1)	0.1615
13 All of **low** back pain patients' problems would be solved if their pain would go away	2.3 (1.5)	2.2 (1.5)	−0.2 (−0.5 to 0.2)	0.3450
**Total HC-PAIRS score (sum of responses to all questions)**	39.6 (10.1)	35.3 (11.7)	−5.6 (−7.6 to −3.6)	<0.0001

For each item, the mean (SD) scores are shown for pre- and post-intervention, the 95% confidence interval for the difference in means and the p-value (paired t-test). In addition, the summation of scores for all questions was calculated to give a total HC-PAIRS score. Lower scores suggest more alignment with current evidence regarding management of patients with low back pain (i.e.; lower scores indicated a movement towards **disagreement** with the questions, which generally suggest that management of patients with low back pain should involve rest rather than activity)

### Self-reported knowledge and skills (gPEP questionnaire)

The pre- to post-intervention change in responses for self-reported knowledge and skills, is shown in Table 3. The general trend for each question is clear from the unmatched data. Some of the questions were left blank by some respondents, which is why the totals vary a little. While approximately 20–35% of responses were inadequate prior to the intervention, only a very small number of people rated their knowledge as inadequate following the intervention (indicating a clear impact of intervention). The paired t-tests showed highly statistically significant movement towards greater knowledge (p<0.0001 for all questions).

**Table 3:** Comparison data for primary care physicians' evidence-based self-reported knowledge and skills.

Self-rating of knowledge and skills regarding:	Pre-intervention n/N (%) inadequate	Post-intervention n/N (%) inadequate	Difference Mean (95% CI)	p-value ^§^
Q1: Current evidence based guidelines (e.g; education, pharmacological and non pharmacologicalinterventions, cognitive behavioural approaches) for the diagnosis and management of acute and chronic low back pain	31/89 (35%)	1/79 (1%)	1.1 (0.9 to 1.3)	<0.0001
Q2: The use of multidisciplinary team-based approaches for people with acute and chronic low back pain	19/89 (21%)	0/80	1.1 (0.9 to 1.3)	<0.0001
Q3: Translating evidence based medicine into your clinical practice for people with acute and chronic low back pain	33/87 (38%)	1/80 (1%)	1.1 (0.9 to 1.3)	<0.0001
Q4: The practical differences between assessment and management of acute and chronic low back pain	22/87 (25%)	1/80 (1%)	1.0 (0.8 to 1.2)	<0.0001
Q5: Similarities and differences in the management of patients presenting to the emergency department with acute low back pain and with an exacerbation of chronic low back pain	26/88 (30%)	2/79 (3%)	0.9 (0.7 to 1.1)	<0.0001
Q6: Importance of and approaches to activity management for people with acute and chronic low back pain	22/88 (25%)	1/80 (1%)	1.1 (0.8 to 1.3)	<0.0001
Q7: Importance of, and approaches to, exercise for people with acute and chronic low back pain	16/88 (18%)	0/80	0.9 (0.7 to 1.2)	<0.0001
Q8: Moderating the impact of acute and chronic low back pain on people, their families and work	19/89 (21%)	1/80 (1%)	0.9 (0.7 to 1.1)	<0.0001
Q9: Pharmacological options for people with acute and chronic low back pain	8/86 (9%)	2/80 (3%)	0.5 (0.3 to 0.8)	<0.0001
Q10: Facilitating the involvement of the patient in the management of acute and chronic low back pain	17/88 (19%)	0/80	0.9 (0.7 to 1.2)	<0.0001
Q11: Health Professionals in your local network that *include* patient active management strategies in their approach to acute and chronic low back pain management	40/88 (45%)	5/80 (6%)	1.1 (0.8 to 1.4)	<0.0001
Q12: Approaches to assist adult learning (such as gPEP being based on self-efficacy theory, pain biology, etc) and facilitating integration of this learning into clinical practice	59/88 (67%)	6/78 (8%)	1.4 (1.1 to 1.6)	<0.0001

The mean difference in paired responses (post- minus pre-intervention) is a measure of change in the raw Likert scores allocated. The positive movement in scores indicates a movement towards clinically adequate (guideline-consistent) responses. ^§^ The p-value is calculated using the paired t-test.

The pre- to post-intervention change in the frequency of recommendations to patients with nsLBP, is shown in Table 4. While this is a little hard to interpret as it is related to PCPs' workload, if the categories are grouped into ‘Never’ and ‘at least once per week’, it becomes evident that there is a general move for questions 13–16 away from the ‘Never’ category. The trend in all of these questions is towards giving this advice or assistance at least some of the time. The kappa statistic for each item was generally low, indicating that the intervention had influenced responses. For most items, the majority of people responding ‘Never’ at the pre-intervention stage responded ‘at least once’ at the final survey.

**Table 4:** Comparison data for primary care physicians' frequency of recommendations for low back pain management.

Frequency per week of strategies recommended for management of patients with non specific low back pain (Pre-intervention response)	N (%)	Post intervention		kappa statistic (95% confidence interval)
		Never	At least once	
C13(a) Advise a patient with acute low back pain to commence a specific exercise program				
Never	11 (13%)	3 (30%)	7 (70%)	0.27 (−0.04 to 0.56)
At least once per week	76 (87%)	4 (6%)	61 (94%)	
C13(b) Advise a patient with chronic low back pain to commence a specific exercise program				
Never	5 (6%)	1 (20%)	4 (80%)	0.32 (−0.16 to 0.79)
At least once per week	80 (94%)	0	69 (100%)	
C14(a) Assist patients with acute low back pain to plan lifestyle changes to improve symptoms				
Never	10 (11%)	3 (38%)	5 (62%)	0.41 (0.06 to 0.77)
At least once per week	77 (89%)	2 (3%)	64 (97%)	
C14(b) Assist patients with chronic low back pain to plan lifestyle changes to improve symptoms				
Never	8 (9%)	3 (43%)	4 (57%)	0.51 (0.14 to 0.88)
At least once per week	77 (91%)	1 (1%)	66 (99%)	
C15(a) Advise patients with acute low back pain on the role of self-management in chronic disease				
Never	14 (16%)	1 (8%)	11 (92%)	0.10 (−0.13 to 0.33)
At least once per week	73 (84%)	1 (2%)	61 (98%)	
C15(b) Advise patients with chronic low back pain on the role of self-management in chronic disease				
Never	10 (12%)	1 (11%)	8 (89%)	0.18 (−0.13 to 0.49)
At least once per week	74 (88%)	0	65 (100%)	
C16 Co-ordinate your management with other health professionals				
Never	8 (10%)	0	5 (100%)	*
At least once per week	73 (90%)	0	62 (100%)	

Response categories were collapsed from four categories into two groups: ‘Never’ and ‘at least once per week’. Only subjects who completed both baseline and follow-up surveys are included in the post-intervention columns of the table. The kappa statistic assessed the degree of change in response (kappa over 0.75 indicates little change, while a low value of kappa indicates that a change has occurred). * kappa cannot be calculated because no respondent marked a ‘Never’ response post-intervention.

The three most important things PCPs obtained from gPEP included the following themes:

1. Management strategies and education (71.5%). Items listed included: a better understanding of pain; increased confidence with managing LBP; improved knowledge of evidence-based assessment and management; making a management plan; pacing advice; importance of belief systems to patient outcome.
2. Importance of an interdisciplinary team approach (51.9%). Items listed included: a multidisciplinary team approach, e.g.; physiotherapy, psychology, exercise program; psychological aspects, including the use of a psychologist; importance of physiotherapy; not all physiotherapists know how to treat LBP.
3. The limitations of imaging (39.5%) and the appropriate use of pharmacological options available/appropriate use of meds (29.6%)

### Clinical practice behaviour (patient vignette)

The pre-post comparison showing PCPs' recommendations for activity, work and bed rest, regarding the acute nsLBP patient vignette, is shown in Table 5. The movement for Q1 was marginally against the recommended guidelines, while the other questions showed movement towards guideline consistent behaviour. Q2 showed a large movement towards guideline consistency. The kappa statistic showed poor agreement (low values), confirming that the responses had changed, and Table 5 shows the direction of the change for each question. While over 85% of respondents who were initially guideline consistent for each item remained consistent, the majority of respondents who were guideline inconsistent for questions 2 and 3 gave guideline consistent responses at post-intervention (82% and 62% respectively). For question 1 (exercise recommendation) the results were less conclusive, with a smaller proportion (39%) of respondents changing from inconsistent to consistent.

**Table 5:** Comparison of primary care physicians' recommendations for acute non specific low back pain management.

Question (Pre-intervention)	N (total = 89)	Post-Intervention guideline-consistent	Post intervention guideline-inconsistent	kappa statistic (95% confidence interval)
**1 Exercise recommendation**				
Guideline consistent	69 (78%)	54 (87%)	8 (13%)	0.47 (0.24 to 0.70)
Guideline inconsistent	20 (22%)	7 (39%)	11 (61%)	
**2 Work recommendation**				
Guideline consistent	53 (60%)	43 (91%)	4 (9%)	0.11 (−0.06 to 0.28)
Guideline inconsistent	36 (40%)	27 (82%)	6 (18%)	
**3 Bed rest recommendation**				
Guideline consistent	66 (74%)	54 (92%)	5 (8%)	0.34 (0.10 to 0.57)
Guideline inconsistent	23 (26%)	13 (62%)	8 (38%)	

For this patient vignette, three statements explored physicians' recommendations regarding exercise, work and bed rest. The percentage of responses that were ‘guideline consistent’ and ‘guideline inconsistent’ at both pre- and post-intervention time points, are shown. Only subjects who completed both baseline and follow-up surveys were included in the post-intervention columns of the table. The kappa statistic assessed the degree of change in response.

The baseline means of the HC-PAIRS questionnaire (total of all questions) were compared between participants classified as ‘guideline consistent’ and ‘guideline non-consistent’ according to the 3 questions on the patient vignette (Table 6). The means for the non-consistent group for each question were significantly higher (p<0.003 for each question) than for the guideline-consistent group.

**Table 6:** Comparison of HC-PAIRS total scores (pre-intervention), between participants classified as ‘guideline consistent’ and ‘guideline inconsistent’.

Question (pre-intervention)	N	Pre-intervention HC-PAIRS (total) mean (SD)	p-value
**1 Exercise recommendation**			
Guideline consistent	18	45.4 (9.2)	0.0024
Guideline inconsistent	62	37.5 (9.5)	
**2 Work recommendation**			
Guideline consistent	33	43.8 (8.6)	0.0005
Guideline inconsistent	47	36.1 (9.6)	
**3 Bed rest recommendation**			
Guideline consistent	20	46.4 (9.1)	0.0001
Guideline inconsistent	60	36.9 (9.1)	

P-values are calculated from the t-test. For each question, the means for the non-consistent group were significantly higher (p<0.003 for each question) than for the guideline-consistent group. A higher HC PAIRS score indicates a stronger belief that pain implies disability and that low back pain should affect daily function, aligning less with the evidence-based recommendations for exercise, work and bed rest.

## Discussion

We demonstrate promising findings in this before/after interprofessional pain education program set within a framework that aligned health policy and practice. Volunteer PCPs were encouraged to adopt more self-reported evidence-based beliefs, attitudes and clinical behaviours for managing their patients with nsLBP. Notwithstanding the lack of control for confounding factors imposed by a cohort design, our data clearly demonstrate a positive impact of the gPEP intervention, with strong evidence for movement of PCPs towards improved beliefs and attitudes regarding LBP, greater self-reported use of evidence based knowledge and clinical skills, and more guideline consistent recommendations for work and activity. Active self management strategies were likely to be recommended more frequently post-intervention. This intervention used an implementation framework which, in our view, may help to bridge a significant gap in the effective translation of evidence into policy and practice.

The significant decrease in HC-PAIRS scores at post-intervention is consistent with improved PCP beliefs and attitudes about pain and impairment in relation to LBP and of a greater magnitude than previously demonstrated for health professionals following an intervention using printed materials only [61]. While the change in total score can be interpreted as clinically important (≥−4.5 points as proposed by Domenech et al [62]), it was also clear that responses to some questions changed more than others. The two questions which changed the most (on average) were: Q2: An increase in pain is an indicator that a low back pain patient should stop what they are doing until the pain decreases; and Q8: Low back pain patients have to be careful not to do anything that might make their pain worse. This shift of almost 1 point on the 7-point Likert scale towards disagreement may reflect the emphasis of gPEP which focused on delivering simple, evidence-based, guideline consistent messages that PCPs could readily convey to their patients. These key messages were consistently repeated throughout the case studies and also linked with specific evidence-based approaches to clinical management, thereby showing the ways in which guidelines could be implemented in a flexible patient-centred manner.

So that PCPs could more easily align their work practices and recognise the potential benefit for patients [63], the case studies were designed to resonate with the guidelines, and improve the coherence with existing PCP consultation practices. In this context, the following guideline consistent recommendations relevant to HC-PAIRS questions 2 and 8, were strongly integrated into our educational material: hurt does not equal harm and some pain is to be expected as you recover from an episode of nsLBP; stay active if possible, moving helps reduce pain; a time-contingent approach to pacing activity can assist in functional recovery from nsLBP; maintain your usual activities; stay at work if possible; avoid prolonged bed rest. Using simple language to convey key messages closely aligns with the approach used in a population-based strategy designed to shift societal views about back pain and which had a sustained effect on PCP beliefs and stated practice behaviour 4.5 years after its cessation [64]. Linking PCP beliefs explicitly to clinical practice behaviours through the use of interactive real clinical cases appears to be a powerful strategy to facilitate adherence to guidelines, potentially more so than printed materials alone [61]. In our case studies, the use of catastrophizing language in patient interactions regarding activity was strongly discouraged, emphasizing the negative influence fear of movement (for example, associated with the catastrophizing or irrational beliefs) on predicted self-reported disability and poor behavioural performance [65]. Other questions from HC-PAIRS (Q3, Q4 and Q9) relating to pain and impairment and aspects of lifestyle moved smaller amounts (approximately 0.5 of a point), and the remaining questions showed no change (Q1, Q5, Q6, Q7, Q10, Q11, Q12 and Q13), probably reflecting the focus of the gPEP intervention towards the key knowledge and skills outlined above.

Scores on the HC-PAIRS have been reported as the only significant predictor of recommendations for work and physical activity (based on a patient vignette) when controlling for possible confounders including gender, years of experience in the treatment of back pain, judgments of severity of symptoms, and judgments of severity of pathology [57]. Our patient vignette data appear consistent with this finding. At baseline, subjects giving guideline inconsistent responses to the vignette questions appeared to gain significantly higher scores on the HC-PAIRS questionnaire (i.e.; more unhelpful beliefs in relation to current evidence regarding LBP and disability). Also, the majority of PCPs who were guideline-inconsistent for work and bed rest recommendations at pre-intervention (40% and 26% respectively), gave guideline-consistent responses at post-intervention, aligning with the shift in HC-PAIRS score. In contrast, the movement for the exercise recommendation was marginally against the recommended guidelines, with a proportion of PCPs changing from guideline consistent to inconsistent. This response may relate to PCPs' interpretation of ‘pacing’, which in gPEP was focused as a time-contingent approach [39] to exercise rather than a pain-contingent approach [66]. Based on the case studies, PCPs were encouraged to adjust activity levels if their patient used a pain-contingent ‘boom-bust’ or ‘overdo-underdo’ approach to exercise and activity (i.e. behavioral modification) and to monitor these changes, especially given that the relationship between fear of movement and function is moderated when pain persists beyond one year [67]. In this regard, a more conservative initial approach to pacing activity in patients with acute nsLBP might be expected, although a graduated increase in exercise and activity would be appropriate in the mid to longer term.

PCPs baseline self-rated knowledge and skills regarding evidence-based aspects of nsLBP varied across the questionnaire items, with the greatest percentage of clinically adequate responses documented for the question which related to the use of pharmacological options for people with nsLBP, as expected for these domain-specific components of medical care (Table 3). In contrast, the highest percentage of clinically-inadequate responses at baseline (excluding item 12 which focused on adult learning), was attributed to the following items: question 11 (45%): health professionals in the local network who include active self-management; question 3 (38%): translating evidence into clinical practice; and question 1(35%): the current evidence based guidelines for the management of nsLBP, reinforcing the need to address these aspects in our intervention. Following gPEP however, an overwhelming movement for all questions towards a more evidence-based approach consistent with a positive benefit from the program was evident, with a very modest percentage (ranging from 1–3% for items 1–9) remaining guideline inconsistent.

The gPEP questionnaire was designed to reflect the knowledge and skills considered essential to effectively implement LBP guidelines into practice and translate this information into clinical behaviours. In this regard, a flexible “whole person” approach similar to that undertaken for our cases studies, may help to moderate some of the clinical tensions associated with matching patient expectation and guideline advice [19]. This approach is possibly reflected here, in the more guideline consistent responses evident post intervention for the gPEP questionnaire. In the case studies, we encouraged PCPs to recognise and directly address concurrent patient factors which may prove to be obstacles to recovery from LBP, including patient perceptions of personal control, the acute/chronic timeline, illness identification and pain self-efficacy [68] (see Case Example S1).

Also the use of screening tools that identify an individual's risk status, and are typically based on predictive psychosocial factors such as catastophizing and depression [69], was undertaken as part of the case studies. These tools included the Orebro Musculoskeletal Pain Screening Questionnaire [45] measuring pain and disability (although the 9 item STarT Back Screening Tool [70] may be more appropriate in primary care), the Depression, Anxiety and Stress Scale (DASS21) measuring emotional functioning [46] and the painDETECT [47] screening for neuropathic pain. Screening tools were perceived as potentially time-efficient for PCPs, and allowing a more comprehensive view of pain and disability, thereby assisting PCPs in planning their management, including the need for engaging other health professionals. Along with a thorough physical examination, and respecting patient preferences, expectations, and previous management, using these screening tools can enable a more comprehensive approach to triaging patients with nsLBP in primary care. A final step in each case study was to outline an evidence-informed, multimodal management plan; one which utilised patient-focused pain education including the use of self-management strategies, pharmacological and behavioural approaches (to movement re-education, time-contingent paced activity and short term goal setting) [30], [71]. Furthermore, we propose that a key factor relevant to the shifts demonstrated on the gPEP questionnaire may relate to the clinical background of the interprofessional team, although we acknowledge that the current research design does not allow us to draw firm conclusions in this regard. Here, the gPEP team reflected a mix of clinicians (physiotherapists, clinical psychologists, rheumatologist) working in primary care (private and hospital settings) alongside specialised clinicians (pain medicine; clinical psychologists and physiotherapists; rheumatologist) working in tertiary facilities (hospitals) and clinical researchers from universities. This primary-tertiary mix, we believe, provided a well balanced and real-world perspective on clinical issues confronted in primary and tertiary care settings and these perspectives were deliberately reflected in the design of the case studies and presented using a ‘shared’ stage, in terms of time and focus.

Collectively, our findings suggest there is a matching of PCP beliefs and clinical practice behaviours. We acknowledge that the self-report measures in this study may not faithfully reflect clinical practice and that patient vignettes have limitations [72], but vignettes are also useful surrogates for understanding PCP approaches to LBP [19], [60], [61]. Clinical case presentations and interactive discussions may potentially help to bridge the gap between evidence and practice, a vital outcome given the alternative to evidence based practice is ‘anything goes’. In this context, gPEP was designed with specific practice enablers to assist PCPs in their main role of delivering an evidence-based patient-centred ‘whole person’ approach to people with nsLBP. Furthermore, the significant shift in the gPEP questions 1, 3, 4, 6, 7 and 8 are consistent with this interpretation, as these questions incorporate specific knowledge and use of practical skills in assessing and managing the multidimensional aspects of nsLBP. An important point to highlight was the bidirectional nature of the gPEP intervention, as the PCPs articulated the practical barriers to implementation such as consultation time constraints for complex pain problems and a lack of funding for integrated interprofessional care.

Furthermore, while at baseline, a small number of PCPs, never recommended a specific exercise program for acute LBP (Table 4), and a small proportion also never recommended self management, regardless of the duration of nsLBP, this was not the case at post-intervention. Following gPEP, the general move in these questions away from the ‘never’ category and towards giving advice or assistance at least some of the time, was in accordance with the patient vignette work and activity recommendations and the favourable shift in HC-PAIRS score. PCPs recommendations for the use of various strategies for managing nsLBP, were made more frequently at post-intervention for the use of a specific exercise program and the use of self management strategies. These data align with the current evidence advocating for the use of active self management as an integral part of the co-care of LBP, and as a result patient outcomes are associated with reduced disability and health care utilisation [30], [37].

The themes that emerged as most important to PCPs following gPEP, were significant in the context of implementing guideline consistent approaches to managing people with nsLBP. In this regard, the reporting of management strategies and patient education are consistent with recommendations from current LBP guidelines [48], [50], [51], [55], [56]. PCPs' improved understanding of the complexity of pain, the importance of belief systems to patient outcomes and their improved knowledge of evidence-based assessment and management all lead to their increased confidence in the skills associated with educating a patient and designing an appropriate management plan. Furthermore, the importance of networking with and using an interdisciplinary team approach as appropriate was highlighted. Given the vast majority of PCPs reported having ‘access to health professionals from other disciplines to assist [with] a team approach to [nsLBP] management’, this clinical network combined with greater confidence in their approach to nsLBP, should facilitate improved health service delivery. However, we acknowledge that the current research design does not allow us to draw firm conclusions regarding the influence of the interprofessional nature of the education intervention as a factor in the positive study outcomes. The appropriate use of psychologists and physiotherapists was frequently mentioned, possibly reflecting the multimodal management approach taken in the case studies, which included a discussion of when to refer to these health professionals and what constituted current best practice by them. The limitations of imaging (39.5%) as a theme was a potentially significant outcome given that imaging is not recommended in over 95% of nsLBP cases, except when red flags are present [50]. Despite this, considerable overuse is still documented [12]. Finally, the pharmacological options available and the appropriate use of medicines for the management of people with nsLBP were noted as important themes. In gPEP, the pharmacological management of people with nsLBP was oriented towards using any therapeutic window as an opportunity for the patient to engage in active self management. We reinforced the importance of taking a multimodal approach to nsLBP management and combining pharmacological approaches with non-pharmacological management including the use of active self management.

Our findings would be strengthened if replicated using a stronger study design such as a randomised controlled trial and expanding the study to include cost and practice effectiveness comparisons made between face-to-face and online educational interventions such as gPEP. Critically, such studies need to include the monitoring of real-world practice behaviours (including PCP referral patterns for prescriptions and radiological imaging and referrals to tertiary facilities). However, it is also clear that the implementation and uptake of clinical guidelines in clinical practice is problematic. This issue highlights the similar factors that complicate such real-world clinical research where time and cost constrain what is possible, sustainable and optimal in high quality research with what is possible, sustainable and optimal for real-world clinicians. Furthermore, the applicability of our findings to other populations of PCPs managing people with persistent LBP may be limited because of the following factors: PCP data were based on self-report measures and these measures may serve to over-estimate the actual change in real practice; responder bias (unlikely here as a good response rate was achieved); and selection bias (PCPs self-referred to gPEP and their motivations for attending this educational intervention may differentiate them from other PCPs). While HC-PAIRS has undergone the most thorough testing to date of any tool for the measurement of health care professionals' attitudes and beliefs to LBP, gaps in the properties of this tool remain, particularly test-retest reliability and responsiveness [73]. The current research design does not allow us to draw firm conclusions regarding the role of the interprofessional nature of the education intervention as a factor in the positive study outcomes.

The adoption of a health policy framework can help when implementing an evidence-based model of care for the management of low back pain in primary care, and has shown promising outcomes in this before/after study with volunteer PCPs. We propose that using a contemporary biopsychosocial perspective of pain combined with evidence-informed knowledge and practical skills delivered by an interprofessional team may be an effective strategy to increase the uptake of clinical guidelines. To better manage the complexities experienced by people with persistent low back pain, we argue for the use of a whole person engagement model. Additionally, both health professionals and people with persistent low back pain may have to modify their expectations from treatment and focus more on the role of care rather than cure.

## Supporting Information

Case Example S1 This case example shows PCPs were encouraged to recognise patient factors relevant to recovery from LBP. PCPs were encouraged to directly address these concurrent factors including patient perceptions of personal control, the acute/chronic timeline, illness identification and pain self-efficacy.
(DOCX)
