# Supplementary material for: A Policy-into-Practice Intervention to Increase the Uptake of Evidence-Based Management of Low Back Pain in Primary Care: A Prospective Cohort Study
Source: PLoS One. 2012 May 25;7(5):e38037. doi: 10.1371/journal.pone.0038037 (PMC3360643; doi:10.1371/journal.pone.0038037)
Supplement: Case Example S1 — This case example shows PCPs were encouraged to recognise patient factors relevant to recovery from LBP. PCPs were encouraged to directly address these concurrent factors including patient perceptions of personal control, the acute/chronic timeline, illness identification and pain self-efficacy. (DOCX) [file pone.0038037.s001.docx]

**Supporting Information File 1**

**S1. This case example shows PCPs were encouraged to recognise patient factors relevant to recovery from LBP.** PCPs were encouraged to directly address these concurrent factors including patient perceptions of personal control, the acute/chronic timeline, illness identification and pain self-efficacy.

For example, if the case study had initially described a person with acute LBP and evidence of fear avoidance behaviors such as hesitancy to bend, PCPs were encouraged to screen the possible reasons for these behaviors (for example, fear of pain; fear of serious pathology; fear of re-injury). The PCP was then encouraged to discuss the strategies that could be used to reduce the threat value of this fear, for example by performing a physical examination (the ‘skill’) to exclude any red flags. This skill then enabled the PCP to reassure the patient (knowledge and clinical decision making) that there was no evidence of serious pathology and therefore, no need for radiological imaging (evidence based practice). If the avoidance behaviours related to movement reflected the patient’s fear of significant pathology, addressing this fear through open rational evidence-informed discussion (knowledge and skills) could be an important first strategy towards the patient adopting a more helpful belief about their LBP. The case discussion would expand to include aspects of simple analgesia that might also facilitate an early active time-contingent approach to activity and movement to manage an acute episode of LBP. Here, PCPs’ knowledge would reflect the evidence base regarding simple analgesia for the management of nsLBP and the necessary skills would include: (i) educating the patient about the use of an appropriate dose of paracetamol to create a therapeutic window of analgesia; (ii) using this window to advise an early paced time-contingent activity (i.e.; prepare a time-contingent pacing approach to pain). These two strategies highlight the need for PCPs to encourage a more active engagement of the patient towards pain co-management, favouring self efficacy, rather than defaulting to a passive pain management approach. If the reasons for fear suggested catastrophising behaviour and hypervigilance, cognitive strategies such as re-framing the threat of pain could be combined with the above strategies in a more formal way (i.e.; consulting a clinical psychologist). While not inclusive of all possible management options, this case example highlights the approach taken in the gPEP intervention with a strong focus on devising shared, interprofessional solutions to optimise patient management.
